# The atherosclerosis of the sinus node artery is associated with an increased history of supra-ventricular arrhythmias: a retrospective study on 541 standard coronary angiograms

**DOI:** 10.7717/peerj.1156

**Published:** 2015-08-27

**Authors:** Michele M. Ciulla, Matteo Astuti, Stefano Carugo

**Affiliations:** 1 Laboratory of Clinical Informatics and Cardiovascular Imaging, Milan, Italy; 2 Department of Clinical Sciences and Community Health, University of Milan, Milan, Italy; 3 Cardiovascular Diseases Unit, Fondazione IRCCS Cà Granda Ospedale Maggiore Policlinico, Milan, Italy; 4 Department of Health Sciences, University of Milan, Milan, Italy

**Keywords:** Sinus node artery, Supraventricular arrhythmias, Atrial fibrillation, Ischemia, Coronary angiography, Atherosclerosis

## Abstract

**Background.** The ischemic damage of the sinus node (SN) is a well known cause of cardiac arrhythmias and can be a consequence of any flow abnormality in the sinus node artery (SNA). Accordingly we aimed this retrospective study to: (1) evaluate the suitability of the standard coronary angiography to study the SNA and (2) determine if the percentage of subjects with a positive retrospective history of supra-ventricular arrhythmias (SVA) differs in patients with normal and diseased SNA ascertained at the time of coronary angiography.

**Methods and Results.** Out of the 541 coronary angiograms reviewed the SNA was visible for its entire course in 486 cases (89.8%). It was found to arise from the right side of the coronary circulation in 266 cases (54.7%) slightly more often than from the left, 219 cases (45.1%). One patient had 2 distinct SNA arising from either side of the coronary circulation. For the second objective, we studied the 333 patients with: (a) coronary artery disease (CAD), (b) properly evaluable SNA and (c) complete clinical history available. In 51 (15.3%) a SNA disease was found, 41.2% of them had a positive SVA history, mainly atrial fibrillation (AF), whereas only 7.4% of patients with a positive history of SVA could be found in the non-SNA diseased. This difference was statistically significant (*P* < 0.001).

**Conclusions.** (1) The evaluation of the SNA is feasible in clinical practice during a standard coronary angiography; (2) this may be relevant since angiographically detectable SNA disease was significantly associated with a positive history of SVA.

## Introduction

The supraventricular arrhythmias, especially AF, have a high prevalence in the general population and, especially in patients with CAD, are a major social and economic burden in terms of mortality and morbidity (Sanoski, 2009). Although many features of their etiology and electrophysiology have already been cleared, some aspects of the SVA pathogenesis have still not been cleared. With the present study, we aimed to explore the possible relationship between a positive retrospective clinical history of SVA and the atherosclerotic disease of the SNA ascertained at the time of coronary angiography. The hypothesis that ischemic damage to the vessels which supply the atrial excito-conducting tissue may impair its function, leading to abnormal heart rhythms, has sound pathophysiological basis. Indeed, the SNA plays an essential role in ensuring the normal operation of SN as a servomechanism (James, 1973), in maintaining its structural integrity and, in particular, that of the collagen matrix that seems to be crucial for the electrical stability of the heart. Furthermore, since the SNA supplies blood to a much larger area than the SN alone, the ischemia secondary to a stenosis of this vessel could give rise to a widespread structural and electrical remodeling, which, as it is known, represents the underlying substrate to most of SVA.

The association between the obstruction of the atrial coronary branches with the occurrence of such arrhythmias during the first phase of a myocardial infarction was already shown several years ago with classical anatomical and imaging studies (Kyriakidis et al., 1992; Tjandrawidjaja et al., 2005; Hod et al., 1987; James, 1961); unfortunately, there are no studies that take into account the effects of the SNA atherosclerosis as a process on the heart rhythm. Therefore, we designed this retrospective study with the purpose to: (1) determine if the standard coronary angiography is a suitable method to assess the SNA by calculating the percentage of angiography where it was possible to detect the SNA; (2) provide further anatomical data about the prevalence of left and right sided SNA in Italy; and (3) evaluate if, in patients with an angiographically detectable and clearly assessable disease of the SNA at the time of coronary angiography, the prevalence of a retrospective clinical history of SVA attributable to a SN hypoperfusion significantly differs from that in patients with a normal SNA.

## Materials and Methods

### Study population

In the present study, we retrospectively analyzed the coronary angiograms performed in the catheterization laboratory of our hospital form May 2013 to May 2014. In those cases where a patient repeated the coronary angiogram procedure, only the older images were taken into account. The coronary angiograms of subjects who had previously undergone bypass surgery were not reviewed. All these exams were suitable for the first and second objective of the study. To achieve the third objective, which focused on the prevalence of the disease the SVA in subjects with and without a previous clinical history of SVA, some exclusion criteria were set up: (a) the subjects in which the SNA could not be entirely and properly evaluated were excluded; this group includes patients with acute coronary syndrome (ACS) where the SNA branched downstream the culprit lesion; (b) the subjects whose clinical report was not available or incomplete were also excluded as those in which intact coronary arteries were detected.

For each patient, the required variables up to the time of the coronary catheterization were extracted from the clinical report of the hospitalization when the coronary angiography was performed; in particular, we carefully collected all the information related to a positive history of SVA and to the presence of known risk factors for CAD and SVA, as well as the main echocardiographic data and any active anti-arrhythmic therapy. To ensure that the experimenters were effectively blinded to the identity of the patients, we have worked on a fully de-identified database containing only the variables in study with a random assignment of an identification code that does not allow investigators to retrieve the identity of the subjects; thus also the need for approval by the IRB was waived.

In the present study, we have included in the SVA all the rhythm disturbances originating from the tissues above the level of the ventricles; furthermore, the atrio-vetricular blocks or conduction delays have not been taken into account as they are a consequence of a lesion of the atrio-ventricular node which does not depend on the SNA for its blood supply. Finally, we excluded the cases where the SVA has arisen for the first time in the early phase of an ACS since the pathogenesis of these acute events is not the object of the present study.

### Angiogaphic analysis

All the coronary angiographies were performed for diagnostic and therapeutic purposes, including both urgent procedures in patients with ACS and elective procedures in patients with stable coronary artery disease or candidates to heart surgery. The selective right and left coronary angiography was performed using a digital cineangiography imaging system (InfinixCS-i; Tokyo, Toshiba, Japan). All images were stored as DICOM data and were analyzed off-line. Since the angiographies were standard procedures, no specific projections were available to study the SNA; thus, all the coronary angiograms were carefully analyzed to assess the SNA, including its presence and origin.

For the third objective of the study, among the examinations where the SNA was fully assessable, those with intact coronary arteries were excluded; however, angiograms showing a subcritical diffuse coronary artery disease or an already-in-place stent were included. Thus, the images were analyzed for the presence of a lesion involving the SNA and the patients were then grouped accordingly. The SNA disease was assessed by applying the standard criteria commonly used for the main subepicardial coronary arteries; not only were critical stenosis taken into account, but also a diffuse irregularity of the arterial wall was considered expression of a pathological vessel. When a chronic lesion of the left circumflex coronary artery (LCX) or the right coronary artery (RCA) was found upstream from the SNA origin, the patient was assigned to the group with SNA disease.

### Statistical analysis

Continuous variables were expressed as median and interquartile ranges. Categorical variables were expressed as absolute numbers and percentages. The differences in continuous variables were assessed using the Mann–Whitney U test when not normally distributed, and the Student t test when normally distributed. The X^2^ test was used to test for differences in categorical variables, unless more than 20% of the cells of the 2 × 2 table had an expected value <5; in these cases, the exact Fisher test was performed. Multivariate analysis was performed to evaluate the relationship between demographic and clinical data. Odds ratios and 95% CI were calculated.

All tests were 2-sided, and a probability value <0.05 was considered statistically significant.

Statistical analyses were performed using SPSS software (version 20, IBM, Armonk, New York, US).

## Results

The size of the sample studies was adequate and the power was calculated (1 − *β* = 0, 9976), with a type I error rate of 5%. Out of the 541 coronary angiograms reviewed, the SNA was detectable and was visible for its entire course in 486 cases (89.8%); it was found to arise from the right side of the coronary circulation in 266 cases (54.7%) slightly more often than from the left, which had 219 cases (45.1%). One patient had 2 distinct SA nodal arteries arising from either side of the coronary circulation (Table 1). Following the exclusion criteria, 67 subjects were excluded since their medical record was not available, 107 for the lack of an angiographically-detectable CAD in the major epicardial arteries, and 34 because the SNA could not be properly evaluated.

**Table 1:** SNA anatomy. Summary of the anatomical characteristics of the SNA visualized in this study.

	No	%
Total number of coronary angiograms reviewed	541	
Entirely visibe SNA	486	89.8
Left-sided SNA	219	45.1
Right-sided SNA	266	54.7
Bilateral SNA	1	0.2

**Figure 1:**
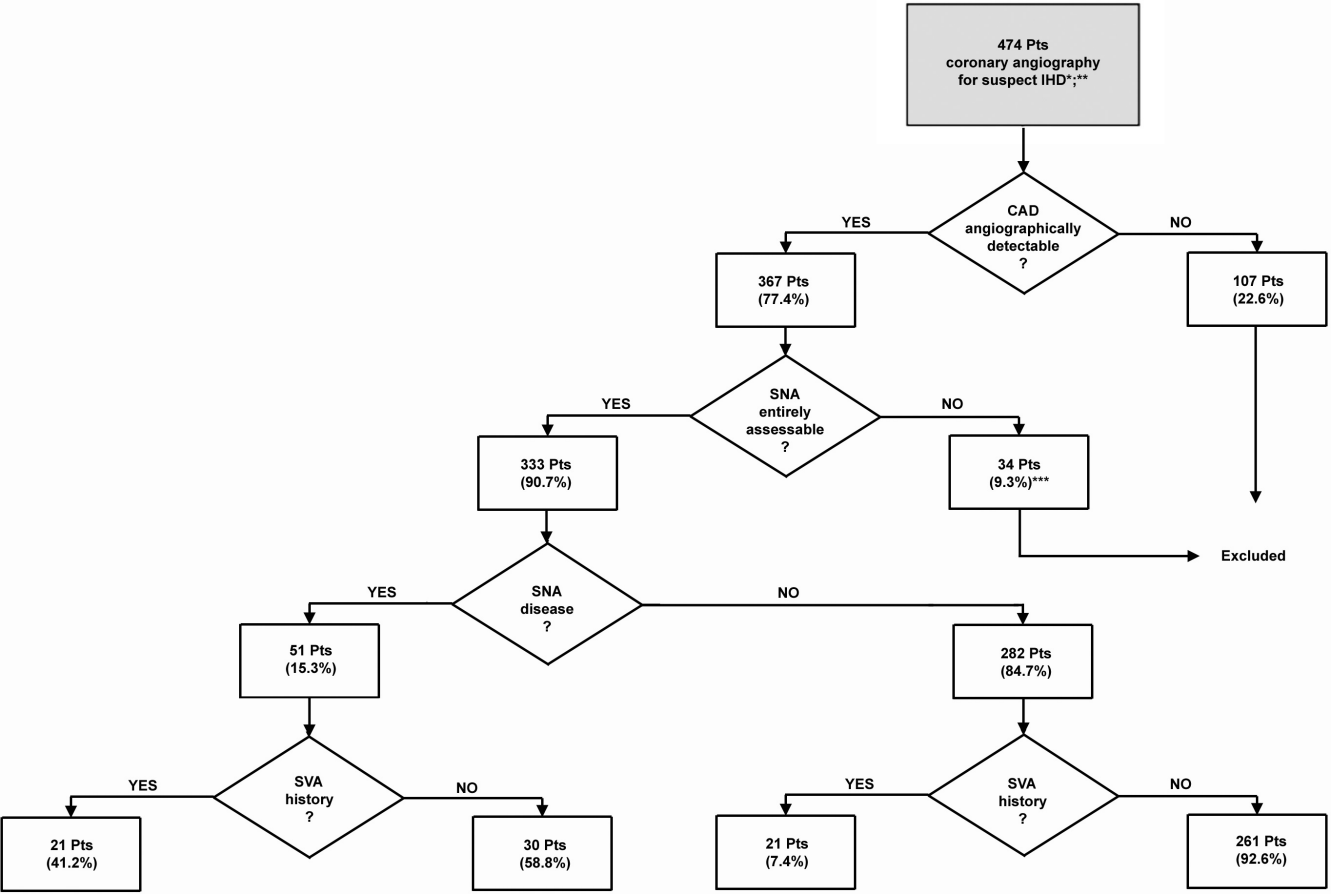
Flow chart showing patient selection according to the exclusion criteria. ∗, patients without medical record were excluded (*n* = 67); ∗∗, only the older angiography was considered for those patients who repeated the exam; ∗∗∗, this group comprehends the images obtained from the primary coronary catheterizations of patients affected by ACS where the SNA branched downstream the culprit lesion.

**Figure 2:**
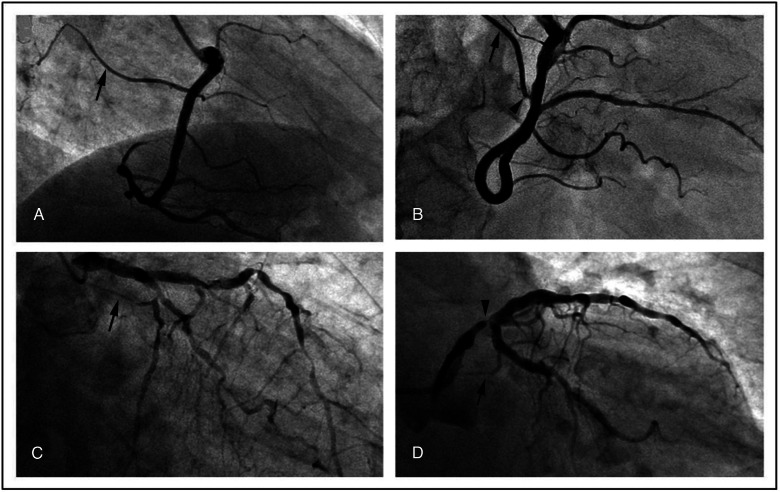
Representative angiographic images of the SNA (arrows) demonstrating the general feasibility of its visualization. (A) a normal SNA; (B) SNA with a focal stenosis (arrowhead); (C) diffuse atherosclerotic disease involving also the SNA; (D) stenosis of the main left coronary artery upstream the SNA (arrowhead).

The remaining 333 were divided into four groups according to the presence/absence of SNA disease and previous clinical history of SVA. The flow chart that shows the selection of patients and the assignment to the study groups is reported in Fig. 1. The clinical profile of each group is shown in Table 2; representative coronary angiograms are shown in Fig. 2. All patient groups were homogeneous in median age and gender. The indication to the coronary catheterization was an ACS in 217 cases (65.2%), 94 (43.3%) of which were ST-segment elevation myocardial infarction. In the remaining cases, the coronary angiography was an elective procedure. No significant differences were found in the distribution of the indication to the coronary catheterization among the groups. Out of the total 333 cases, in 51 (15.3%) a SNA disease was found, in 26 cases the lesion consisted of a diffuse irregularity of the SNA wall, in 19 cases it was a focal stenosis of the SNA, and in the remaining 6 cases the lesion was located in the LCX or RCA upstream from the SNA origin (see Fig. 2). The prevalence of a previous clinical history of SVA was significantly higher in the SNA diseased group than in the non-SNA diseased (41.2% vs. 7.4 % *P* < 0.001) (Table 2). When taking into consideration the classification of SVA reported and their distribution according to the presence/absence of SNA disease, no significant differences where found (Table 3). The prevalence of known risk factors for CAD and SVA and of the cardiovascular therapies was homogeneous among all the groups with the following exceptions: the subjects with SNA disease had a significantly higher prevalence of prior acute myocardial infarction (AMI) (43.1% vs. 23.8%; *P* = 0.004), a significantly lower left ventricle ejection fraction (51.0% vs. 36.2% ; *P* = 0.045) and a significantly higher use of beta-blockers (47.1% vs. 31.2% ; *P* = 0.027) and thienopyridines (23.5% vs. 12.4%; *P* = 0.036) than non-SNA diseased. As expected, in subjects with a clinical history of SVA, irrespective of the presence of SNA disease, a higher prevalence of risk factors for arrhythmia, including a higher prevalence of reduced left ventricle ejection fraction and atrial dilatation, was found alongside with a significantly higher consumption of antiarrhythmic drugs (Table 2).

**Table 2:** Characteristics of the studied patients. Tables patients groups and characteristics. *P* < 0.05 is considered statistically significant.

Characteristics		Total	SNA disease			No SNA disease			*P* values			
			Total	SVA+	SVA-	Total	SVA+	SVA-	P1	P2	P3	P3c
Patients groups	No	333	51	21	30	282	21	261				
	%	100	15.3	41.2	58.8	84.7	7.4	92.6				
Age (yr)	Median	68	73	77	69.5	67	74	66	0.052	0.059	0.058	0.178
	IR	60–78	61–80.5	70–82	57–78	59–76	70–78	58–76				
Female sex	No	88	11	5	6	77	6	71	0.744	0.892	0.393	0.251
	%	26.4	21.6	23.8	20.0	27.3	28.6	27.2				
ACS (STEMI)	No	217 (94)	32 (12)	13 (3)	19 (9)	185 (82)	10 (3)	175 (79)	0.917	0.071	0.693	0.438
	%	65.2 (43.3)	62.7 (37.5)	61.9 (23.1)	63.3 (47.4)	65.6 (44.3)	47.6 (30.0)	67.0 (45.1)				
Hypertension requiring treatment	No	237	42	20	22	195	15	180	0.640	0.814	0.055	0.732
	%	71.2	82.4	95.2	73.3	69.1	71.4	69.0				
Prior AMI	No	89	22	10	12	67	3	64	0.589	0.425	**0.004**	**0.025**
	%	26.7	43.1	47.6	40.0	23.8	14.3	24.5				
DM	No	75	11	3	8	64	2	62	0.490	0.178	0.859	0.627
	%	22.5	21.6	14.3	26.7	22.7	9.5	23.8				
PAD	No	89	18	9	9	71	7	64	0.344	0.371	0.133	0.604
	%	26.7	35.3	42.9	30.0	25.2	33.3	24.5				
**Smoke**									0.189	0.221	0.693	0.097
Current	No	89	14	3	11	75	3	72				
	%	26.7	27.5	14.3	36.7	26.6	14.3	27.6				
Former	No	84	15	8	7	69	8	61				
	%	25.2	29.4	38.1	23.3	24.5	38.1	23.4				
Never	No	160	22	10	12	138	10	128				
	%	48.1	43.1	47.6	40.0	48.9	47.6	49.0				
Left ventricle ejection fraction <55%	No	128	26	12	14	102	13	89	0.461	**0.011**	**0.045**	0.756
	%	38.4	51.0	57.1	46.7	36.2	61.9	34.1				
**Atrial dilatation:**									**0.014**	**0.017**	0.431	0**.**319
Na	No	145	18	7	11	127	7	120				
	%	43.4	35.3	33.3	36.7	45.0	33.3	46.0				
No	No	110	19	4	15	91	4	87				
	%	33.0	37.3	19.1	50.0	32.3	19.0	33.3				
Yes (mild)	No	78 (45)	14 (11)	10 (7)	4 (4)	64 (34)	10 (2)	54 (32)				
	%	23.4 (57.7)	27.4 (78.6)	47.6 (70)	13.3 (100)	22.7 (53.1)	47.7 (20)	20.7 (59.3)				
Cholesterol level: total (mg/dl)	Median	172	166	172	153	172	149	173	0.393	**0.05**	0.302	0.103
	IR	143.5–200.5	143–190	155–191	136–186	144–204.5	136–180	145–208				
Cholesterol level: HDL (mg/dl)	Median	43	42	42	39.5	43	43.5	43	0.153	0.986	0.337	0.123
	IR	35.5–52	35.5–49.5	38–50	32–48	36–52	37–53	35–52				
TGL (mg/dl)	Median	115	105	110	104	117	95	119	0.916	**0.038**	0.379	0.073
	IR	82–153.5	83–149	95–145	79–153	82–155	63–131	88–159				
Hyperthyroidism	No	6	2	0	2	4	1	3	0.506	0.267	0.230	0.431
	%	1.8	3.9	0.0	6.7	1.4	4.8	1.1				
**Renal function, creatinine clearance (ml/min)**									0.092	**0.004**	0.108	0.711
CKD stage 0–1, ≥90	No	78	9	1	8	69	2	67				
	%	23.4	17.6	4.8	26.7	24.5	9.5	25.7				
CKD stage 2, ≥60 to 89	No	163	24	9	15	139	12	127				
	%	48.9	47.1	42.8	50.0	49.3	57.1	48.6				
CKD stage 3, ≥30 to 59	No	79	13	8	5	66	6	60				
	%	23.7	25.5	38.1	16.7	23.4	28.6	23.0				
CKD stage 4, ≥15 to 29	No	12	5	3	2	7	0	7				
	%	3.6	9.8	14.3	6.6	2.5	0.0	2.7				
CKD stage 5, <15	No	1	0	0	0	1	1	0				
	%	0.3	0.0	0.0	0.0	0.3	4.8	0.0				
ACE inhibitor/ARB	No	163	30	10	20	133	13	120	0.174	0.160	0.125	0.347
	%	48.9	58.8	47.6	66.7	47.2	61.9	46.0				
Amiodarone	No	7	3	3	0	4	2	2	0.064	**0.029**	0.076	0.534
	%	2.1	5.9	14.3	0.0	1.4	9.5	0.8				
Beta-blocker	No	112	24	11	13	88	9	79	0.524	0.231	**0.027**	0.524
	%	33.6	47.1	52.4	43.3	31.2	42.9	30.3				
Digoxin	No	3	1	1	0	2	2	0	0.412	**0.005**	0.394	0.667
	%	0.9	2.0	4.8	0.0	0.7	9.5	0.0				
Calcium blocker	No	59	10	3	7	49	7	42	0.495	0.067	0.701	0.986
	%	17.7	19.6	14.3	23.3	17.4	33.3	16.1				
ASA	No	156	26	10	16	130	9	121	0.688	0.757	0.520	0.889
	%	46.8	51.0	47.6	53.3	46.1	42.9	46.4				
Thienopyridine	No	47	12	4	8	35	1	34	0,739	0,489	**0,036**	0.264
	%	14.1	23.5	19.0	26.7	12.4	4.8	13.0				
IC antiarrhythmic drugs	No	7	3	3	0	4	4	0	0.064	**<0.001**	0.076	0.665
	%	2.1	5.9	14.3	0.0	1.4	19.0	0.0				
Statin	No	110	19	8	11	91	7	84	0.917	0.914	0.486	0.847
	%	33.0	37.3	38.1	36.7	32.3	33.3	32.2				
Sotalol	No	2	1	1	0	1	0	1	0.421	1	0.283	0.345
	%	0.6	2.0	4.8	0.0	0,4	0.0	0.4				

**Notes.**

SVA + subjects with a positive history of SVA SVA − subjects without a positive history of SVA P1 comparison between subjects affected by SNA disease with and without SVA P2 comparison between subjects not affected by SNA disease with and without SVA P3 comparison between subjects with and without SNA disease P3c correction for multiple testing Na not assessable

**Table 3:** Classification of SVA. Distribution of the different kinds of SVA observed according to the presence or not of SNA disease.

Supraventricular arrhythmias		All patients	SNA disease	No SNA disease
Total	No. (%)	42 (100)	21 (50)	21 (50)
AF		35 (83.3)	17 (81)	18 (85.7)
Paroxysmal AF		9 (21.4)	5 (23.8)	4 (19)
Persistent AF		12 (28.6)	7 (33.3)	5 (23.8)
Permanent AF		14 (33.8)	5 (23.8)	9 (42.9)
Sick sinus syndrome		3 (7.1)	3 (14.3)	0 (0)
SV PACs requiring antiarrhythmic therapy		2 (4.8)	0 (0)	2 (9.5)
Paroxysmal supraventricular tachycardia		2 (4.8)	1 (4.8)	1 (4.8)

## Discussion

With the present study, we demonstrated that the evaluation of the vessel deputed to vascularization of the SN is feasible without any particular effort during a standard coronary angiography. We found that the SNA could be entirely visualized in 89.8% of the examined cases. Previous studies aimed to visualize the SNA by using the same technique have reported an higher percentage of visualization (Al-Shanafey et al., 2001). This difference can be explained by the fact that those exams were specifically carried out to identify the SNA; this was not the case of the present study, which is based on coronary angiograms performed with projections aimed to visualize and, eventually, treat lesions on the major epicardial coronary arteries. In this regard, we think that the real life data provided by this study may have a more immediate impact on the clinical practice suggesting to check when feasible the patency of the SNA during a standard coronary angiography, especially in the presence of a history of SVA. Furthermore, we provide some additional anatomic information regarding the left rather than right-sided origin of the SNA with the latter found to be slightly more frequent (57.7% vs. 45.1%), confirming the previous findings of older studies in European, North American and Brazilian subjects (Cezlan et al., 2012; Saremi et al., 2008; Ortale, Paganoti & Marchiori, 2009). In our experience, the angiographic projection to visualize the SNA vary depending on the inconstant position and course of this vessel; however, we have observed that a right-sided SNA can be better visualized with a Right Anterior Oblique (RAO) straight (−30°; 0°) or a Left Anterior Oblique (LAO) cranial (+45°; +20°) view, whereas a LAO caudal “spider” view (+45°; −30°) or a RAO caudal view (−20°; −20°) are the most suitable to show a left-sided SNA branching from the proximal LCX.

**Figure 3:**
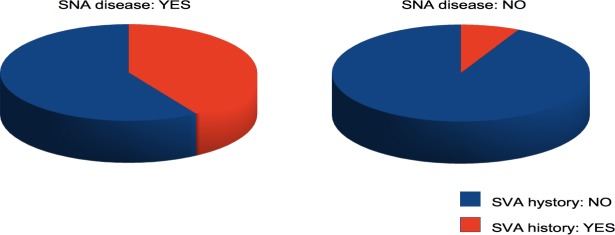
Pie chart showing the prevalence of SVA history in subjects with and without SNA disease.

In addition, we wanted to explore the relationship between heart rhythm and atherosclerosis of the SNA. It has already been demonstrated that SVA, and in particular AF, are associated with an increased prevalence of CAD (Nucifora et al., 2009) and even subclinical CAD (ESC, 2013); in the present study, we wanted to better delineate the specific role of the SNA disease at the time of coronary angiography in this context. Therefore, the most relevant finding of this study was that, among 333 patients with angiographically detectable CAD, the prevalence of a retrospective clinical history of SVA, mainly AF, was significantly higher in the subjects with a diseased SNA (Fig. 3). The fact that also the prevalence of prior AMI and of reduced left ventricle ejection fraction is significantly more elevated in the same group, and consequently an increased beta-blockers and thienopyridine antiplatelets usage, may be a consequence of a more severe CAD with an increased probability of the involvement of minor coronary branches such as the SNA. Furthermore, the difference in myocardial contractile function cannot be considered a confounding factor; in fact, although it is a well-known risk factor for SVA, this pro-arrhythmic action involves an increase in telediastolic ventricular pressure and a consequent increased atrial pressure and volume overload that leads to atrial dilatation and stretch, but in the present study the prevalence of atrial dilatation did not significantly differ between subjects with and without SNA disease.

Different studies were carried on to investigate the possible relationship between SVA, mainly AF, atherosclerosis in general, and with coronary arteries atherosclerosis in particular. A cohort study demonstrated that the risk of AF was associated with carotid intima-media thickness (IMT) and severity of carotid plaques (Heeringa et al., 2007). A further study confirmed the association between lone AF with increased carotid IMT and arterial stiffness. In this case, arterial stiffness was found to be higher in persistent than paroxysmal lone AF patients suggesting that a gradation of subclinical vascular disease can be associated with a parallel gradation of the severity of AF (Chen et al., 2013).

A case-control study found a higher prevalence of obstructive CAD, assessed by Multislice Computed Tomography Coronary Angiography among patients with paroxysmal or persistent AF (Nucifora et al., 2009). On the other hand, the specific role of SNA disease in the genesis of SVA was also explored, but in limited situations. Previous angiographic (Kyriakidis et al., 1992; Tjandrawidjaja et al., 2005; Hod et al., 1987) and anatomical (James, 1961) studies succeeded in demonstrating the relation between an acute occlusion of the atrial coronary branches, including the SNA, and the onset of SVA in the early stages of AMI. Finally, angiographically detectable SNA disease was significantly associated with AF post-coronary artery bypass graft surgery (Al-Shanafey et al., 2001; Kolvekar et al., 1997).

The present study is a step in the understanding of the complex role of atherosclerotic disease and CAD in the genesis of supra-ventricular rhythm disturbances for different reasons. First, we demonstrated a statistically significant association between SNA disease at the time of coronary angiography and the prevalence of a retrospective clinical history of SVA attributable to a SN hypoperfusion, in a context different from the acute phase of an ACS and in a population of patients wider than the candidates for by-bass surgery. Second, the results of this study suggest an interesting physiopathological mechanism of chronic SVA, even if the design of this study does not allow us to draw any conclusion regarding a causal relationship between the disease of the SNA and SVA. Nevertheless, we have already explained the different pathophysiological ways through which a lesion that determines a flow abnormality in the SNA may affect the heart rhythm; that said, we do not think that SNA disease is necessary, although it may be sufficient, to provoke a SVA, but it should be regarded as one of the SVA risk factors along with arterial hypertension, heart failure, valvular disease and so on.

This study has some limitations that should be acknowledged. First, it is a case-control study, the limitations of which are well known. A larger study, with follow-up data, may provide more conclusive information. Second, this study is limited by attempting to correlate angiographic anatomy rather than ischemia per se, but there is currently no reliable means of assessing atrial ischemia. Third, angiographic images are the current gold standard to show alterations of the arterial lumen, but they cannot give definite information regarding the their atherosclerotic nature. An IVUS study should be more conclusive in this sense, but it is currently unavailable for such a narrow vessel as the SNA. These limitations do not diminish the value of the result of this study, which provides new data supporting the association between the disease of a specific branch of the coronary circulation and a positive history of SVA.

## Supplemental Information

10.7717/peerj.1156/supp-1 Supplemental Information 1 Dataset Raw dataset.
